# Pyroxsulam Resistance in *Apera spica-venti*: An Emerging Challenge in Crop Protection

**DOI:** 10.3390/plants14010074

**Published:** 2024-12-29

**Authors:** Soham Bhattacharya, Madhab Kumar Sen, Katerina Hamouzová, Pavlína Košnarová, Rohit Bharati, Julio Menendez, Josef Soukup

**Affiliations:** 1 Department of Agroecology and Crop Production, Faculty of Agrobiology, Food and Natural Resources, Czech University of Life Sciences Prague, Kamýcká 129, 165 00 Prague, Czech Republic; bhattacharya@af.czu.cz (S.B.); kosnarova@af.czu.cz (P.K.); soukup@af.czu.cz (J.S.); 2 Plant Virus and Vector Interactions, Crop Research Institute, Drnovská 507, 161 06 Prague, Czech Republic; 3 Departamento de Ciencias Agroforestales, Escuela Politécnica Superior, Campus Universitario de La Rábida, 21071 Palos de la Frontera, Huelva, Spain; jmenend@dcaf.uhu.es

**Keywords:** ALS-inhibiting herbicide, cytochrome P450s, GSTs, non-target-site resistance, selection pressure

## Abstract

*Apera spica-venti*, a prevalent weed in Czech winter wheat fields, has developed resistance to ALS-inhibiting herbicides due to their frequent use. This study reports a biotype of *A. spica-venti* resistant to pyroxsulam, with cross and multiple resistance to iodosulfuron, propoxycarbazone, pinoxaden, and chlortoluron. Dose–response experiments revealed high resistance of both R1 and R2 biotypes to pyroxsulam, with resistance factors (RF) of 6.69 and 141.65, respectively. Pre-treatment with malathion reduced RF by 2.40× and 1.25× in R1 and R2, indicating the potential involvement of cytochrome P450 (CytP450). NBD-Cl pre-treatment decreased RF only in R2, suggesting possible GST involvement. Gene analysis revealed no mutations (at previously reported sites) or overexpression in the acetolactate synthase (*ALS*) gene. However, a significant difference in ALS enzyme activity between resistant and susceptible biotypes points to target-site resistance mechanisms. Studies with ^14^C-labeled pyroxsulam showed that reduced absorption and translocation were not likely resistance mechanisms. In summary, herbicide resistance in *A. spica-venti* appears to result from multiple mechanisms. Possible causes include target-site resistance from an unidentified *ALS* mutation (within coding or regulatory regions). Enhanced herbicide metabolism via CytP450s and GSTs is also a contributing factor. Further experimental validation is needed to confirm these mechanisms and fully understand the resistance. This evolution underscores the adaptive capacity of weed populations under herbicide pressure, emphasizing the need for alternative control strategies.

## 1. Introduction

*Apera spica-venti* (L.) P. Beauv. (loose silky bentgrass) is considered one of the most important weeds in winter cereals in the Czech Republic as well as in neighboring European countries [1]. This monocotyledonous annual grass is mainly found in arable land areas with moderate and temperate agroclimatic conditions [2]. This troublesome weed is rapidly evolving resistance, mainly due to its well-adapted lifecycle to winter cereals, which can cause yield losses of up to 30-40% in oilseed rape, forage crops, and early sown summer cereals [3].

To date, it has evolved resistance against three modes of herbicide across eleven different countries (https://www.weedscience.org/Pages/Species.aspx, accessed on 2 June 2024). Several studies have shown that *A. spica-venti* is considered a significant grass weed, especially in Northern, Eastern, and Central Europe [2,4]. Moreover, Babineau et al. (2017) also suggested that silky bentgrass quickly evolves resistance to several herbicides (especially acetolactate synthase (ALS), acetyl CoA carboxylase (ACCase), and photosystem II inhibitors), mainly due to its high genetic variability [4]. Additionally, Massa et al.’s (2013) model (encompassing all of Europe) indicated that crop rotations with winter cereals and the use of conventional tillage systems might substantially elevate the risk of selecting for *A. spica-venti* resistance [5]. In the Czech Republic, the management of this noxious weed has become increasingly challenging, particularly with ALS-inhibiting herbicides like pyroxsulam, due to its rapidly evolving resistance [1,6]. Cases of herbicide resistance in *A. spica-venti* have also been identified in New Zealand [7], Lithuania [8], Germany [9], and Poland [10].

Since their appearance on the global plant protection market, ALS-inhibiting herbicides have gained increasing popularity as chemical weed control measures. Due to their intensive and repeated use, Due to their heavy and repeated use, ALS inhibitors are the herbicides most commonly associated with weed resistance [11]. Herbicide resistance in plants can occur through different mechanisms, such as target-site resistance (TSR) and non-target-site resistance (NTSR). Point mutations in the gene sequence of the target protein and target gene amplification are key mechanisms of TSR in grass weeds [12,13]. On the other hand, the NTSR mechanisms encompass enhanced metabolism, reduced herbicide uptake or translocation, and vacuolar sequestration [14]. The main enzymes involved in NTSR are cytochrome P450 monooxygenases (CytP450s), glutathione S-transferases (GSTs), glycosyltransferases, ATP-binding cassette (ABC) transporters, etc. [13,15,16]. Detecting enhanced metabolism can be very challenging, particularly due to its intricate network, making it difficult for farmers to manage. Unfortunately, many modern and frequently used herbicide active ingredients, such as sulfonylureas or triazolopyrimidines, are prone to metabolism [17].

CytP450-mediated enhanced metabolism has been reported in many species like *Bromus sterilis* [18], *Lolium rigidum* [19], and *Alopecurus myosuroides* [20]. Farmers in the Czech Republic have lately observed the resistance of *A. spica-venti* to recommended doses of pyroxsulam in winter wheat fields. The management of this resistant weed has become difficult due to the limited herbicidal alternatives available. Hence, the current study investigates the mechanisms of herbicide resistance, focusing on the roles of point mutations, gene overexpression, and detoxification enzymes such as CytP450 and GSTs. Point mutations in herbicide target genes and the overexpression of detoxifying enzymes are suspected to contribute to resistance, allowing plants to degrade herbicides and survive treatment. Additionally, the study explores the possibility of cross and multiple resistance to other herbicides. This research will help fill a critical knowledge gap and lay the groundwork for future studies using omics-based approaches at the molecular level.

## 2. Materials and Methods

### 2.1. Plant Materials and Growth Conditions

The resistant (R) seeds were collected from two distinct winter wheat fields near Jindřichův Hradec, Czech Republic [(49.0119139 N, 14.7266231 E) and (49.0978289 N, 14.7354286 E)]. These biotypes are referred to as R1 and R2, respectively. Farmers in these areas had previously noted the low efficacy of pyroxsulam. The susceptible biotype (S) was collected from the Jindřichův Hradec region, Czech Republic (48.9609217 N, 14.7383469 E). All the collected seeds were kept in the dark at room temperature until their further use. To account for variability in field conditions, seed samples were collected from at least 100 plants in the same field. The pot experiment was conducted in an open-air vegetation hall with an attached rooftop to avoid the rain. Seeds were sown in (10 seeds per pot) 343 cm^3^ plastic pots filled with chernozem soil [high fertility property and moisture storage capacity, clay content 46% (loamy soil), soil pH (potassium chloride) 7.5, sorption capacity of soil 209 mmol (+), 87 mg kg^−1^ phosphorus, 203 mg kg^−1^ potassium, 197 mg kg^−1^ magnesium, 8073 mg kg^−1^ calcium]. Seedlings were regularly watered and fertilized as per requirements.

### 2.2. Dose–Response Assay

A dose–response experiment was conducted between March and May 2023 with four replicates per treatment. Pyroxsulam was applied at the two-to-three-leaf stage at rates of 4.6875, 9.375 (recommended field rate), 18.75, 37.5, 75, 150, and 300 g a.i. (active ingredient) ha^−1^ for R biotypes and 0.29296875, 0.5859375, 1.171875, 2.34375, 4.6875, and 9.375 g a.i. ha^−1^ for S biotypes using a laboratory spray chamber equipped with a Lurmark 015F80 nozzle calibrated to a spray volume of 250 L ha^−1^ and a pressure of 120 kPa. The herbicide efficacy was evaluated 28 days after treatment by comparing biomass reduction in treated pots with the untreated control based on dry weight.

In a separate experiment, the inhibitors malathion (Malathion, PESTANAL^®^, analytical standard, Sigma-Aldrich, Merck Group, St Louis, MO, USA) and 4-chloro-7-nitrobenzoxadiazole (NBD-Cl, Sigma-Aldrich, Merck Group, St Louis, MO, USA) were applied at rates of 1000 g a.i. ha^−1^ and 270 g a.i. ha^−1^, respectively. Malathion and NBD-Cl were diluted in water and applied 1 h and 48 h before pyroxsulam treatment. The same pyroxsulam rates as in the dose–response experiment (9.375, 18.75, 37.5, 75, 150, and 300 g a.i. ha^−1^) were used in this experiment. Additionally, malathion and NBD-Cl were each applied individually at the same rates without pyroxsulam to assess their effects. The efficacy of these treatments was also evaluated 28 days after application by comparing biomass reduction based on dry weight. These experiments were performed according to Han et al. (2024) [21].

### 2.3. Cross- and Multiple-Resistance Experiments with Other Herbicides

Four different herbicides were used for cross-resistance and multiple-resistance studies. Propoxycarbazone and iodosulfuron were used (applied at 42 and 40 g a.i. ha^−1^, respectively) for the cross-resistance study. Chlortoluron (PS II inhibitor) and pinoxaden (ACCase inhibitor) were used (applied at 1000 and 45 g a.i. ha^−1^, respectively) for the multiple-resistance study. These experiments were conducted with four replicates for each treatment, and the efficacy was estimated 28 days after treatment by comparing biomass reduction in the treated pot with the untreated control based on the dry weight.

### 2.4. In Vitro ALS Activity Assay

A bulk sample of 2 g leaf tissues from the S and R *A. spica-venti* biotypes was collected from 4-week-old plants for the ALS activity assay. ALS enzyme extraction and in vitro activity assays were performed according to Hamouzová et al. (2011) [2]. Pyroxsulam was used as an active ingredient with a concentration of 10^−14^ to 10^−4^ nM. Specific enzyme activity was estimated using a standard curve and was expressed as µmol of acetoin mg^−1^ protein h^−1^.

### 2.5. Partial ALS Gene Substitution and Overexpression Studies

*ALS* mutation studies were conducted using 35 individuals from each population that survived the highest herbicide dosages in the dose–response studies, targeting mutation sites known to be associated with resistance to ALS-inhibiting herbicides [22]. Approximately 80 mg of shock-frozen leaf tissues from both R and S biotypes were collected for genomic DNA (gDNA) extraction using the DNeasy Plant Mini Kit (QIAGEN, Hilden, Germany), following the manufacturer’s instructions. Moreover, fresh leaf tissues of the same quantity per sample were also used for total RNA extraction using the RNeasy Mini Kit (QIAGEN, Hilden, Germany), following the manufacturer’s instructions. Subsequently, complementary DNA was synthesized utilizing the High-capacity cDNA Reverse Transcription Kit (Applied Biosystems, Foster City, CA, USA). Primers were designed (Table 1) based on publicly available sequences of the *ALS* gene from *A. spica-venti* (JN646110.1) using Primer-BLAST and Primer3 software.

A C1000 thermocycler (Bio-Rad, Hercules, CA, USA) was used for polymerase chain reaction (PCR) with 50 ng of total gDNA per reaction. The PCR cycling profile was programmed as an initial denaturation step at 95 °C for 5 min followed by 40 cycles of 5 s at 95 °C, 10 s at 56 to 63 °C (based on the annealing temperature of the primers), and 30 s at 72 °C, along with a final extension step for 10 min at 72 °C. Amplified PCR products were separated on 1.8% agarose gel, and purification was performed using a GeneJET Gel Extraction Kit (Thermo Scientific, Waltham, MA, USA) as per the manufacturer’s instructions. Finally, the purified gel products were sent for DNA sequencing (Eurofins Genomics Germany GmbH, Ebersberg, Germany).

For *ALS* gene expression analysis, plant samples were treated with pyroxsulam, and samples were collected 24 h after treatment. The qRT-PCR assay was performed using PowerUp SYBR Green Master Mix (Applied Biosystems, USA) with the StepOne™ Real-Time PCR System (Applied Biosystems, USA). The reaction mixture consisted of 5 μL of SYBR Green Master Mix, 1 μL of primer mix, and 4 μL of gDNA (7.5 ng/μL) or cDNA (7.5 ng/μL). The thermocycling conditions included an initial denaturation at 95 °C for 5 min followed by 40 cycles of 15 s at 95 °C and 1 min at 57–60 °C, depending on the primer annealing temperature. Melting curves were generated by gradually increasing the temperature from 60 °C to 95 °C. *TBP* and *GAPDH* were selected as reference genes, as determined to be appropriate for *A. spica-venti* based on the study by Wrzesińska et al. (2021) [23]. Each qRT-PCR experiment was conducted with five biological replicates. The quantification cycle threshold (Ct) values obtained using the StepOne system were exported for further analysis.

### 2.6. ^14^C Pyroxsulam Absorption and Translocation Study

*A. spica-venti* R and S plants were treated with ^14^C pyroxsulam solutions mixed with a pyroxsulam commercial formulation on the adaxial surface of the second leaf. A PB-600 micro-applicator (Hamilton Company, Reno, NV, USA) was utilized for herbicide application at a rate of 9.375 g a.i. ha^−1^ with a spraying volume of 250 L ha^−1^ for pyroxsulam. Each plant received four 0.5 μL droplets of this mixture on the adaxial surface of the leaf, with a specific activity of 0.54 kBq μL^−1^. Plants were maintained in the growth chamber under the growing conditions until evaluation. The ^14^C pyroxsulam absorption and translocation study was conducted according to the previously described method by Palma-Bautista et al. (2023) [24]. The evaluation of translocation and absorption was performed at 12, 24, 48, 72, and 96 h after treatment (HAT). The radioactivity of the absorbed and non-absorbed pyroxsulam and combustions was analyzed by liquid scintillation spectrometry (LSS). The rate of recovery, absorption, and translocation of ^14^C herbicide was calculated according to Palma-Bautista et al. (2021) [25]. The percentage of ^14^C herbicide recovery was greater than 85% in all samples assayed.

### 2.7. Statistical Analysis

R program software (version: 4.1.0) was used to analyze the data from the dose-response assay as described by Sen et al. (2021) [18]. The three-parameter log-logistic function where the lower limit is equal to 0 was used for parameter estimates.

*y* = *d*/(1 + exp (*b* (log (*x*) − log (GR_50_)))) (1)

where *y* is the dependent variable (efficacy, weight, etc.), *x* is the dose, *d* is the upper limit, *b* is the slope, and GR_50_ is the value at which the biotype exhibited growth reduction by 50%. The 50% growth inhibition by herbicide dose, or GR_50_, values were calculated for each herbicide and each population. Based on this, resistance factors (RFs) were calculated by dividing the GR_50_ of the R population by the GR_50_ of the S population. For the cross- and multiple-resistance studies, the chosen herbicides were applied as per the recommended dose, and the results were estimated by one-way analysis of variance (ANOVA) in the R-Studio program. Comparisons among the values were performed based on Tukey’s HSD test (*p* < 0.05).

## 3. Results

### 3.1. Whole-Plant Dose–Response Experiments

The whole-plant dose–response experiment proved that both biotypes evolved resistance against pyroxsulam (Table 2). The GR_50_ of the S biotype was 1.1 g a.i. ha^−1^, whereas the GR_50_s of the R1 and R2 biotypes were 7.5 and 158.4 g a.i. ha^−1^. However, pre-treatment with malathion (1000 g a.i. ha^−1^) caused a 2-fold decrease in the resistance factor (RF) in the R1 (RF: 2.8) and a 1.3-fold decrease for the R2 (RF: 112.7) biotype when compared to values for plants treated with only pyroxsulam (Table 2). Correspondingly, a reduction in RF was noted in the plants of the R2 biotype, with an RF value of 81.8 when the plants were pre-treated with NBD-Cl. However, applying malathion or NBD-Cl alone at the same rate did not reduce biomass in any of the biotypes (Table S1).

### 3.2. Cross and Multiple Resistance to Other Herbicides

**Figure 1:**
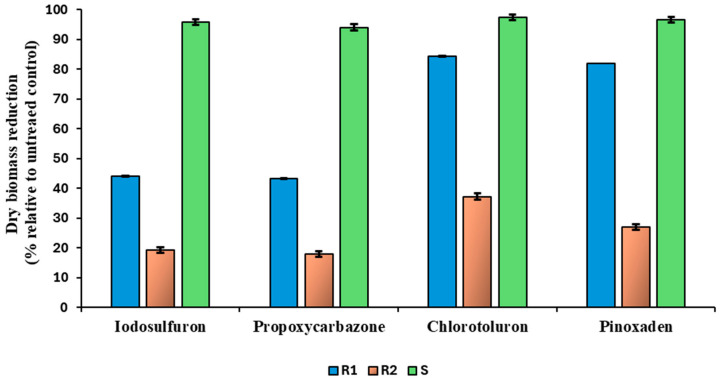
Dry biomass reduction (% relative to untreated control) of the resistant (R) and susceptible (S) biotypes of *A. spica-venti* for cross- and multiple-resistance studies. Dry biomass reduction percentages between R and S biotypes are presented, showing that S biotypes exhibit significantly greater biomass reduction compared to R biotypes under herbicide treatment. These results are based on the average dry biomass of each biotype after herbicide treatment (see Figure S1).

Based on the dry weight of surviving plants, pyroxsulam-resistant biotypes were highly resistant against other groups of ALS-inhibiting herbicides. We found that both R1 and R2 biotypes were resistant to iodosulfuron, with an average dry biomass reduction of 44.15% and 19.23%, respectively, and to propoxycarbazone, with an average dry biomass reduction of 43.29% and 17.945%, respectively. In contrast, the S biotype showed an average dry biomass reduction of 95.72% and 94.01%, respectively, after treatment with each herbicide (Figure 1 and Figure S2). Propoxycarbazone belongs to the triazolopyrimidine group, whereas iodosulfuron belongs to the sulfonylurea group. Similarly, both R1 and R2 biotypes were resistant against pinoxaden, with an average dry biomass reduction of 81.81% and 26.92%, respectively. At the same time, the S biotype showed an average dry biomass reduction of 96.58%. The R2 biotype was also found to be resistant to the PSII-inhibiting herbicide chlortoluron, with an average dry biomass reduction of 37.17% (Figure 1 and Figure S2).

### 3.3. ALS Enzyme Activity

**Figure 2:**
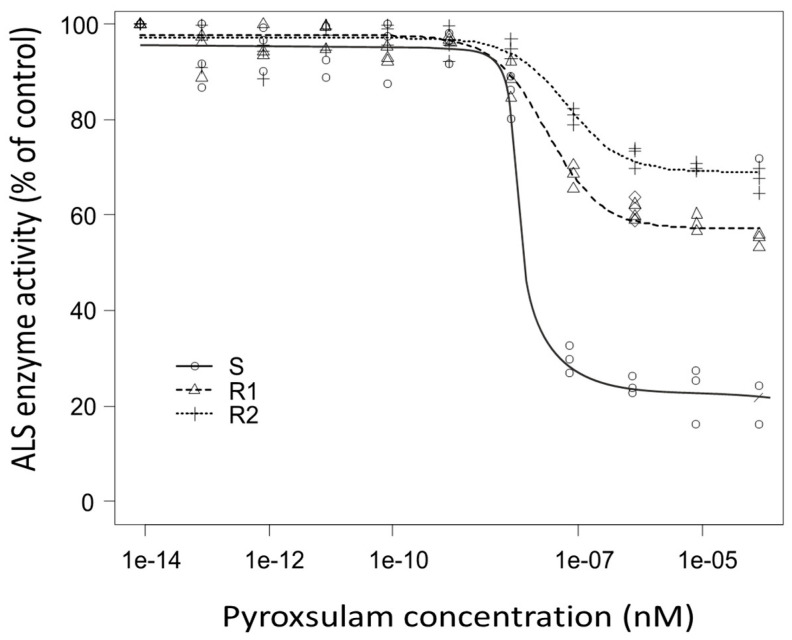
ALS enzyme activity of *A. spica-venti* populations in response to pyroxsulam.

For a further confirmation of resistance, ALS enzyme activity was compared between S and R biotypes. In the absence of pyroxsulam, no differences in ALS activity were observed among the tested biotypes. However, upon the addition of pyroxsulam, the ALS protein activity of the R biotypes was higher compared to that of the S biotype (Figure 2). A higher concentration of pyroxsulam was required to inhibit ALS enzyme activity in the R biotypes compared to that for the S biotype. The R1 and R2 biotypes both displayed IC_50_ values higher than the highest dose tested (>10^−4^ nM).

### 3.4. ALS Gene Mutation and Expression Analysis

**Figure 3:**
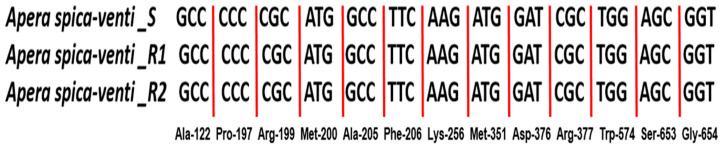
Partial ALS gene sequencing results of *A. spica-venti* R and S biotypes. The numbers are based on *A. thaliana ALS*.

Partial *ALS* gene sequencing was performed using different primers to identify the TSR based on *ALS* gene mutations for specific sites such as Ala-122, Pro-197, Arg-199, Met-200, Ala-205, Phe-206, Lys-256, Met-351, Asp-376, Arg-377, Trp-574, Ser-653, and Gly-654 (according to *Arabidopsis thaliana ALS*). In the current study, none of the biotypes revealed any mutations for the above-mentioned sites (Figure 3).

**Figure 4:**
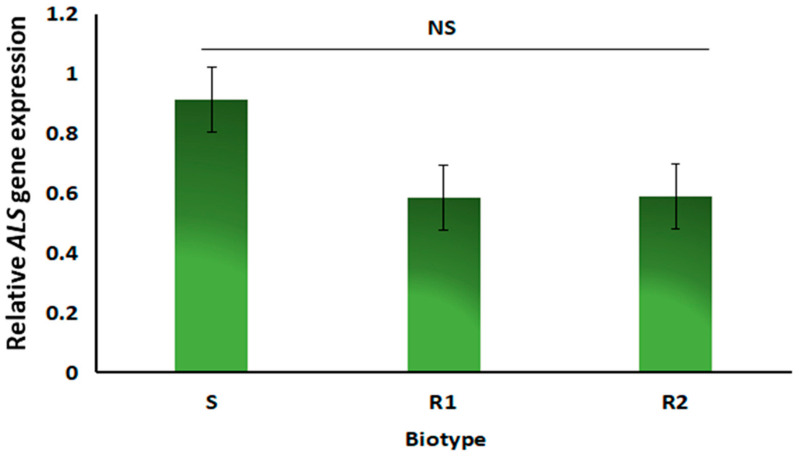
*ALS* gene expression level. The results were compared based on a two-sample *t*-test at a 5% significance level. NS represents not significant.

The *ALS* gene expression study using qRT-PCR revealed no difference in the *ALS* gene expression between the R biotypes and the S biotype (Figure 4). Therefore, our results indicated that *ALS* gene mutations and overexpression might not have contributed to resistance against pyroxsulam in the *A. spica-venti* population.

### 3.5. Pyroxsulam Translocation and Penetration

At 24 HAT, a higher percentage of pyroxsulam absorption was observed in the S biotype (41.1%) compared to that in the R2 (27.4%) biotypes. But after that, the pyroxsulam absorption rate was found to be similar in all tested biotypes at 48, 72, and 96 HAT, and a considerable amount of pyroxsulam was found inside the treated plants (37.8–57.4%) (Table 3).

Therefore, the absorption difference at 24 HAT could not be justified as a reduced absorption of the herbicide. For translocation, more than 90% of the penetrating herbicide was found in the treated leaves for all biotypes. Thus, a lower translocation of pyroxsulam was exhibited in *A. spica-venti*. At 12 HAT, an herbicide translocation difference was observed between S and R biotypes in systemic leaves and roots. The S biotype had a higher translocation percentage in systemic leaves (4.1%) compared to that in R1 (1.6%) and R2 (1.1%). Similarly, in the roots, the S biotype showed a translocation percentage of 3.5%, while R1 and R2 exhibited 0.6% and 0.5%, respectively, but as the herbicide percentage was very negligible compared to the herbicide found in the treated leaves, this difference could not support translocation as a possible herbicide resistance mechanism.

## 4. Discussion

In the current study, we report two pyroxsulam-resistant *A. spica-venti* populations from the Czech Republic. To the best of our knowledge, this represents the first instance of NTSR-based pyroxsulam resistance in any common windgrass species, although further validation might be needed. In a previous study, Košnarová et al. (2021) reported an *A. spica-venti* from the Czech Republic with a high resistance to pyroxsulam (RF = 269.4); however, they found target-site mutations (Pro-197-Thr and Trp-574-Leu mutations) for pyroxsulam [1]. Contrary to that study, in the current study, the RF values are much lower (40.3× in R1 and 1.9× in R2, as compared to the previous study). However, a significant decrease in the RF was detected upon malathion (in both R1 and R2) and NBD-Cl (only in the case of R2) pre-treatment. Dose–response experiments following pre-treatment with malathion and NBD-Cl have been utilized as indicators of metabolism-based herbicide resistance in various studies, including a study on *Papaver rhoeas* [26].

The combined observations from the earlier study by Košnarová et al. (2021) and the current study highlight the dynamic nature of herbicide resistance evolution and emphasize the need for ongoing monitoring and research [1]. This shift indicates that the resistant common windgrass populations are likely adapting in response to selection pressure, potentially through metabolic detoxification pathways rather than solely through target-site mutations [27]. In addition to pyroxsulam, resistance against pinoxaden, iodosulfuron, and propoxycarbazone was also detected. The cross and multiple resistance (in our case) may be ascribed to the heightened activity of herbicide detoxification mechanisms through metabolic enzymes [28,29]. A similar outcome was observed in pyroxsulam-resistant *B. sterilis* from the Czech Republic, where enhanced metabolism contributed to resistance mechanisms and conferred resistance to other groups of ALS-inhibiting herbicides [18].

Additionally, the resistant biotypes showed lower ALS enzyme inhibition with pyroxsulam doses compared to the sensitive ones. This increased activity is most likely due to a target-site resistance (TSR) mechanism, potentially involving an unidentified mutation in the ALS enzyme that reduces the herbicide’s effectiveness [30]. Alternatively, the resistance could result from increased post-transcriptional stability of the enzyme, allowing it to remain functional for a longer period in resistant biotypes, independent of gene expression levels or copy number variations [31]. However, further experimental validation will be needed to confirm these hypotheses and draw definitive conclusions. Nevertheless, multiple mechanisms of herbicide resistance have been documented in many weed species [32,33].

No evidence of ALS mutations at previously reported sites or of overexpression, differences in the herbicide penetration rates, or reductions in pyroxsulam translocation were detected. To date, to the best of our knowledge, there has been only one report of the reduced translocation of pyroxsulam as a resistance mechanism [34], but reduced absorption and translocation have been identified in many other weed species [13,35,36,37]. Additionally, our study found that CytP450s and GSTs might be involved in the enhanced metabolism of the herbicide, contributing to the overall resistance. However, further omics-based research is needed to confirm the differential CytP450 and GST expression to elucidate specific mechanisms involved in resistance. Studies examining herbicide metabolites, detoxification rates, and the complete detoxification pathway are also essential to provide a comprehensive understanding. Omics data, including genomics, transcriptomics, and metabolomics, are well-known for their crucial roles in elucidating various biological processes and traits in weeds. In the current context, these omics approaches might offer valuable insights and novel information.

## 5. Conclusions

This study demonstrates that *A. spica-venti* populations in the Czech Republic exhibit resistance to pyroxsulam, along with cross and multiple resistance to iodosulfuron, propoxycarbazone, pinoxaden, and chlortoluron. As an underlying mechanism, no known *ALS* gene mutations or overexpression were detected, although resistant biotypes exhibited increased ALS enzyme activity. The resistance appears to be enhanced herbicide metabolism, likely mediated by CytP450s and GSTs, which contribute to the rapid detoxification of herbicides. However, the possibility of additional resistance mechanisms, including unidentified mutations in the ALS enzyme or other biochemical pathways, requires further investigation to fully elucidate the underlying molecular processes. Based on our observations, we recommend that farmers stop using pyroxsulam, pinoxaden, and other easily metabolized herbicides in this area. We also suggest incorporating herbicide mixtures with more durable modes of action and diversifying weed management strategies, such as crop rotation, mechanical control, and delayed sowing. These steps can reduce weed populations, lower selection pressure, and slow resistance development, offering more effective and sustainable control.

## Figures and Tables

**Table 1:** List of primers used for *ALS* gene sequencing and expression analysis.

Primer Name	Sequence (5′ to 3′)		Amplicon Size (in bp)	Annealing Temperature (in °C)	Mutation Points Covered (Numbers According to *Arabidopsis thaliana*)
Forward primer (F1)	ATGGCCACAGCCACGTCCAC		710	62.8	Ala-122, Pro-197, Arg-199, Met-200, Ala-205, Phe-206
Reverse primer (R1)	CCTCTACTATGGGCGTCTCC				
Forward primer (F2)	TCTGTATGTTGGTGGTGGCT		266	59.6	Met-351, Asp-376, Arg-377
Reverse primer (R2)	CAATCTTGGACCTGCTTGCA				
Forward primer (F3)	TGATGGGGATGGTAGCTTCC		409	56.9	Trp-574, Ser-653, Gly-654
Reverse primer (R3)	TTAATAAGAAACCCTGCCAT				
Primers for quantitative real-time PCR	ALS_ Forward primer	CACAACTACCTGGTCCTCGA	166	57	_
	ALS_ Reverse primer	ATCCTGGCAGACTCATTGGA			
	GAPDH_ Forward primer	CAGTCACTGTCTTCGGTGTCA	150		
	GAPDH_ Reverse primer	GCAGAGATGACCACCTTCTTG			
	TBP_ Forward primer	GGCTCTTGTGATGTCAAATTTCC	126	58.7	
	TBP_ Reverse primer	GAACAATCTTCGGTTGCTTCA			

**Table 2:** Results of the pyroxsulam dose–response experiment for resistant (R) and susceptible (S) biotypes.

Chemical Ingredients	Biotype	b (±SE)	d (±SE)	GR_50_ (±SE)	*p*-Value	RF
Pyroxsulam	R1	0.62 (±0.2)	98.97 (±5.7)	7.5 (±2.3)		6.7
	R2	0.78 (±0.1)	98.4 (±3.6)	158.4 (±26.7)		141.7
	S	0.73 (±0.04)	95.92 (±6.5)	1.1 (±0.2)		NA
Malathion + pyroxsulam	R1	0.53 (±0.1)	100 (±5.1)	3.1 (±2)	<2e-16	2.8
	R2	0.76 (±0.1)	99.1 (±3.2)	127.5 (±11.9)	0.0085	112.7
	S	0.83 (±0.04)	98.02 (±4.7)	1.1 (±0.1)	0.10	NA
NBD-Cl + pyroxsulam	R1	0.41 (±0.1)	98.58 (±9.5)	5.4 (±2.6)	0.93	5.2
	R2	0.84 (±0.1)	97.83 (±2.4)	84.3 (±8.6)	0.0384	81.8
	S	0.82 (±0.04)	96.55 (±4.5)	1 (±0.1)	0.77	NA

‘GR_50_’ is the rate of herbicide (g a.i. ha^−1^) required to reduce shoot dry weight by 50%, ‘b’ is the slope around the GR50, ‘d’ is the upper limit, ‘SE’ represents the standard error, and ‘resistance factor (RF)’ is calculated as resistant/susceptible based on GR_50_ ratios. NA indicates not applicable. *p*-value represents a significant difference in GR_50_ between pyroxsulam vs. malathion + pyroxsulam and NBD-Cl + pyroxsulam.

**Table 3:** Herbicide penetration and translocation of pyroxsulam. Different letters within the same column indicate significant differences (Tukey’s HSD test, *p* < 0.05). The data are presented as mean ± standard deviation.

Time Duration	Biotype	Penetration %	Translocation %		
		Average (±SD)	Leaves (±SD)	Systemic Leaves (±SD)	Roots (±SD)
12 HAT	S	44.9 (±4.5) a	92.4 (±4.7) a	4.1 (±2.1) a	3.5 (±2.5) b
	R1	39.8 (±1.8) a	97.8 (±0.5) a	1.6 (±0.4) b	0.6 (±0.2) a
	R2	37 (±9.1) a	98.4 (±0.1) a	1.1 (±0.1) b	0.5 (±0.0) a
24 HAT	S	41.1 (±5.4) bc	96.3 (±0.9) a	1.8 (±0.4) a	1.9 (±0.7) a
	R1	38.8 (±5) b	93.3 (±2.8) a	4.5 (±1.8) a	2.2 (±1.3) a
	R2	27.4 (±2.0) ab	92.2 (±1.3) a	5.1 (±1.2) a	2.7 (±0.5) a
48 HAT	S	40.0 (±5.5) a	96.4 (±0.6) a	2.5 (±0.4) a	1.1 (±0.2) a
	R1	47.3 (±7.4) a	97.6 (±0.8) a	1.5 (±0.5) a	0.9 (±0.4) a
	R2	37.8 (±4.7) a	96.3 (±1.0) a	2.5 (±0.7) a	1.2 (±0.3) a
72 HAT	S	55.5 (±6.2) a	95.4 (±1.9) a	3.5 (±1.6) a	1.1 (±0. 4) a
	R1	57.5 (±7.2) a	97.4 (±0.4) a	2.0 (±0.4) a	0.6 (±0.1) a
	R2	48.0 (±7.0) a	94.9 (±1.4) a	3.3 (±0.8) a	1.8 (±0.6) a
96 HAT	S	58.0 (±4.718) a	95.5 (±1.6) a	3.6 (±1.4) a	0.8 (±0.2) a
	R1	48.31 (±5.2) a	95.4 (±1.5) a	3.5 (±1.1) a	1.1 (±0.4) a
	R2	53.4 (±4.0) a	97.5 (±0.9) a	1.8 (±0.6) a	0.7 (±0.3) a

## Data Availability

The dataset is available on request from the authors.

## Supplementary Materials

The following supporting information can be downloaded at: https://www.mdpi.com/article/10.3390/plants14010074/s1, Table S1. Average dry weight biomass of *Apera spica-venti* biotypes for control ± untreated), herbicide, herbicide+inhibitor, and inhibitor alone treatment at the recommended dose; Figure S1. Fitted logarithmic dose-response curves for the *A. spica-venti* after application of (A) pyroxsulam, (B) pyroxsulam + malathion, and (C) pyroxsulam + NBD-Cl; Figure S2. The dry biomasses of resistant (R) and the susceptible (S) biotype of *A. spica-venti* for cross and multiple resistance studies. “UT” refers to untreated and “T” refers to treated.

## References

[1] Košnarová P., Hamouz P., Hamouzová K., Linn A., Sen M.K., Mikulka J., Soukup J. (2021). *Apera spica-venti* in the Czech Republic Develops Resistance to Three Herbicide Modes of Action. Weed Res..

[2] Hamouzová K., Soukup J., Jursík M., Hamouz P., Venclová V., Tůmová P. (2011). Cross-Resistance to Three Frequently Used Sulfonylurea Herbicides in Populations of *Apera spica-venti* from the Czech Republic. Weed Res..

[3] Massa D., Krenz B., Gerhards R. (2011). Target-Site Resistance to ALS-Inhibiting Herbicides in *Apera spica-venti* Populations Is Conferred by Documented and Previously Unknown Mutations. Weed Res..

[4] Babineau M., Mathiassen S.K., Kristensen M., Holst N., Beffa R., Kudsk P. (2017). Spatial Distribution of Acetolactate Synthase Resistance Mechanisms in Neighboring Populations of Silky Windgrass (*Apera spica-venti*). Weed Sci..

[5] Massa D., Kaiser Y.I., Andújar-Sánchez D., Carmona-Alférez R., Mehrtens J., Gerhards R. (2013). Development of a Geo-Referenced Database for Weed Mapping and Analysis of Agronomic Factors Affecting Herbicide Resistance in *Apera spica-venti* L. Beauv. (Silky Windgrass). Agronomy.

[6] Hamouzová K., Košnarová P., Salava J., Soukup J., Hamouz P. (2014). Mechanisms of Resistance to Acetolactate Synthase-Inhibiting Herbicides in Populations of *Apera spica-venti* from the Czech Republic. Pest Manag. Sci..

[7] Hulme P.E. (2024). Potential Risks of Future Herbicide-Resistant Weeds in New Zealand Revealed through Machine Learning. N. Z. J. Agric. Res..

[8] Auškalnienė O., Kadžienė G., Stefanovičienė R., Jomantaitė B. (2020). Development of Herbicides Resistance in *Apera spica-venti* in Lithuania. Zemdirbyste-Agric..

[9] Schulz A., Mathiassen S.K., de Mol F. (2014). Approaches to Early Detection of Herbicide Resistance in *Apera spica-venti* Regarding Intra- and Inter-Field Situations. J. Plant Dis. Prot..

[10] Adamczewski K., Matysiak K. (2012). The Mechanism of Resistance to ALS-Inhibiting Herbicides in Biotypes of Wind Bent Grass (*Apera spica-venti* L.) with Cross and Multiple Resistance. Pol. J. Agron..

[11] Heap I. The International Herbicide-Resistant Weed Database. Online. Friday, 15 December 2023. www.weedscience.org.

[12] Torra J., Alcántara-De La Cruz R. (2022). Molecular Mechanisms of Herbicide Resistance in Weeds. Genes.

[13] Gaines T.A., Duke S.O., Morran S., Rigon C.A.G., Tranel P.J., Küpper A., Dayan F.E. (2020). Mechanisms of Evolved Herbicide Resistance. J. Biol. Chem..

[14] De la Cruz R.A., Da Silva Amaral G., Mendes K.F., Rojano-Delgado A.M., De Prado R. (2020). Absorption, Translocation, and Metabolism Studies of Herbicides in Weeds and Crops. Radioisotopes in Weed Research.

[15] Goldberg-Cavalleri A., Onkokesung N., Franco-Ortega S., Edwards R. (2023). ABC Transporters Linked to Multiple Herbicide Resistance in *Alopecurus myosuroides*. Front. Plant Sci..

[16] Wang J., Cao W., Guo Q., Yang Y., Bai L., Pan L. (2022). Resistance to Mesosulfuron-Methyl in *Beckmannia syzigachne* May Involve ROS Burst and Non-Target-Site Resistance Mechanisms. Ecotoxicol. Environ. Saf..

[17] Dimaano N.G., Iwakami S. (2021). Cytochrome P450-Mediated Herbicide Metabolism in Plants: Current Understanding and Prospects. Pest Manag. Sci..

[18] Sen M.K., Hamouzová K., Mikulka J., Bharati R., Košnarová P., Hamouz P., Soukup J. (2021). Enhanced Metabolism and Target Gene Overexpression Confer Resistance Against Acetolactate Synthase-Inhibiting Herbicides in *Bromus sterilis*. Pest Manag. Sci..

[19] Gaines T.A., Lorentz L., Figge A., Herrmann J., Maiwald F., Ott M.-C., Han H., Busi R., Yu Q., Powles S.B. (2014). RNA-Seq Transcriptome Analysis to Identify Genes Involved in Metabolism-Based Diclofop Resistance in *Lolium rigidum*. Plant J..

[20] Délye C., Gardin J.A.C., Boucansaud K., Chauvel B., Petit C. (2011). Non-Target-Site-Based Resistance Should Be the Centre of Attention for Herbicide Resistance Research: *Alopecurus myosuroides* as an Illustration. Weed Res..

[21] Han Y., Gao H., Sun Y., Wang Y., Yan C., Ma H., Huang Z. (2024). Target Gene Overexpression and Enhanced Metabolism Confer Resistance to Nicosulfuron in *Eriochloa villosa* (Thunb.). Pestic. Biochem. Physiol..

[22] Ghanizadeh H., Buddenhagen C.E., Griffiths A.G., Harrington K.C., Ngow Z. (2024). Target-Site and non-target site resistance mechanisms are associated with iodosulfuron resistance in *Lolium perenne* L. N. Z. J. Agric. Res..

[23] Wrzesińska B., Kościelniak K., Frąckowiak P., Praczyk T., Obrępalska-Stęplowska A. (2021). The Analysis of Reference Genes Expression Stability in Susceptible and Resistant *Apera spica-venti* Populations under Herbicide Treatment. Sci. Rep..

[24] Palma-Bautista C., Vázquez-García J.G., de Portugal J., Bastida F., Alcántara-De La Cruz R., Osuna-Ruiz M.D., Torra J., De Prado R. (2023). Enhanced Detoxification via Cyt-P450 Governs Cross-Tolerance to ALS-Inhibiting Herbicides in Weed Species of *Centaurea*. Environ. Pollut..

[25] Palma-Bautista C., Vázquez-García J.G., Domínguez-Valenzuela J.A., Ferreira Mendes K., Alcántara De La Cruz R., Torra J., De Prado R. (2021). Non-Target-Site Resistance Mechanisms Endow Multiple Herbicide Resistance to Five Mechanisms of Action in *Conyza bonariensis*. J. Agric. Food Chem..

[26] Torra J., Rojano-Delgado A.M., Menéndez J., Salas M., De Prado R. (2021). Cytochrome P450 Metabolism-Based Herbicide Resistance to Imazamox and 2,4-D in *Papaver rhoeas*. Plant Physiol. Biochem..

[27] Délye C. (2013). Unravelling the Genetic Bases of Non-Target-Site-Based Resistance (NTSR) to Herbicides: A Major Challenge for Weed Science in the Forthcoming Decade. Pest Manag. Sci..

[28] Yu Q., Powles S. (2014). Metabolism-Based Herbicide Resistance and Cross-Resistance in Crop Weeds: A Threat to Herbicide Sustainability and Global Crop Production. Plant Physiol..

[29] Beckie H.J., Tardif F.J. (2012). Herbicide Cross Resistance in Weeds. Crop Prot..

[30] Alcántara-De La Cruz R., Rojano-Delgado A.M., Giménez M.J., Cruz-Hipolito H.E., Domínguez-Valenzuela J.A., Barro F., De Prado R. (2016). First Resistance Mechanisms Characterization in Glyphosate-Resistant *Leptochloa virgata*. Front. Plant Sci..

[31] Zhong V., Archibald B.N., Brophy J.A. (2023). Transcriptional and Post-Transcriptional Controls for Tuning Gene Expression in Plants. Curr. Opin. Plant Biol..

[32] Hada Z., Menchari Y., Rojano-Delgado A.M., Torra J., Menéndez J., Palma-Bautista C., Souissi T. (2021). Point Mutations as Main Resistance Mechanism Together with P450-Based Metabolism Confer Broad Resistance to Different ALS-Inhibiting Herbicides in *Glebionis coronaria* from Tunisia. Front. Plant Sci..

[33] Palma-Bautista C., Vázquez-García J.G., Osuna M.D., Garcia-Garcia B., Torra J., Portugal J., De Prado R. (2022). An Asp376Glu Substitution in *ALS* Gene and Enhanced Metabolism Confers High Tribenuron-Methyl Resistance in *Sinapis alba*. Front. Plant Sci..

[34] Sen M.K., Hamouzová K., Onkokesung N., Menendez J., Torra J., Košnarová P., Sellamuthu G., Gupta A., Bharati R., Sur V.P. (2023). Transcriptomic Response in Pyroxsulam-Resistant and Susceptible *Bromus sterilis* Identified Three Distinct Mechanisms of Resistance. bioRxiv.

[35] Souza A.d.S., Leal J.F.L., Montgomery J.S., Ortiz M.F., Simões Araujo A.L., Morran S., de Figueiredo M.R.A., Langaro A.C., Zobiole L.H.S., Nissen S.J. (2023). Nontarget-Site Resistance Due to Rapid Physiological Response in 2,4-D Resistant *Conyza sumatrensis*: Reduced 2,4-D Translocation and Auxin-Induced Gene Expression. Pest Manag. Sci..

[36] Nandula V.K., Ray J.D., Ribeiro D.N., Pan Z., Reddy K.N. (2013). Glyphosate Resistance in Tall Waterhemp (*Amaranthus tuberculatus*) from Mississippi Is Due to Both Altered Target-Site and Nontarget-Site Mechanisms. Weed Sci..

[37] Nalin D., Munhoz-Garcia G.V., Witter A.P.W., Takeshita V., Oliveira C., Adegas F.S., Tornisielo V.L., Oliveira Junior R.S.D., Constantin J. (2023). Absorption, Translocation, and Metabolism of Glyphosate and Imazethapyr in Smooth Pigweed with Multiple Resistance. Agronomy.

