# Supplementary material for: Pyroxsulam Resistance in Apera spica-venti: An Emerging Challenge in Crop Protection
Source: Plants (Basel). 2024 Dec 29;14(1):74. doi: 10.3390/plants14010074 (PMC11722645; doi:10.3390/plants14010074)
Supplement: Supplementary file 1 [file plants-14-00074-s001.zip › plants-3352623-supplementary.pdf]

Article

Pyroxsulam Resistance in *Apera spica-venti*: An Emerging Challenge in Crop Protection

Soham Bhattacharya [1], Madhab Kumar Sen [1,*], Katerina Hamouzová [1,*], Pavlína Košnarová [1], Rohit Bharati [2], Julio Menendez [3] and Josef Soukup [1]

[1] Department of Agroecology and Crop Production, Faculty of Agrobiology, Food and Natural Resources, Czech University of Life Sciences Prague, Kamýcká 129, 165 00 Prague, Czech Republic; bhattacharya@af.czu.cz (S.B.); kosnarova@af.czu.cz (P.K.); soukup@af.czu.cz (J.S.)

[2] Plant Virus and Vector Interactions, Crop Research Institute, Drnovská 507, 161 06 Prague, Czech Republic; rohit.bharati@vurv.cz

[3] Departamento de Ciencias Agroforestales, Escuela Politécnica Superior, Campus Universitario de La Rábida, 21071 Palos de la Frontera, Huelva, Spain; jmenend@dcaf.uhu.es

* Correspondence: senm@af.czu.cz (M.K.S.); hamouzova@af.czu.cz (K.H.)

Table S1. Average dry weight biomass of *Apera spica-venti* biotypes for control ±untreated), herbicide, herbicide + inhibitor, and inhibitor alone treatment at the recommended dose

Biotype	Active ingredient	Dose (g a.i. Ha-1)	Average dry biomass weight ± SD
R 1	untreated	0	0.58 ± 0.10
R 2	untreated	0	0.39 ± 0.04
S	untreated	0	0.59 ± 0.07
R 1	Pyroxsulam	9.375	0.20 ± 0.05
R 2	Pyroxsulam	9.375	0.41 ± 0.03
S	Pyroxsulam	9.375	0.09
R 1	Pyroxsulam+ malathion	9.375+ 1000	0.12 ± 0.07
R 2	Pyroxsulam+ malathion	9.375+ 1000	0.37 ± 0.06
S	Pyroxsulam+ malathion	9.375+ 1000	0.02
R 1	Pyroxsulam+ NBD-Cl	9.375+ 270	0.18 ± 0.01
R 2	Pyroxsulam+ NBD-Cl	9.375+ 270	0.31 ± 0.09
S	Pyroxsulam+ NBD-Cl	9.375+270	0.01
R 1	Malathion	1000	0.57 ± 0.11
R 2	Malathion	1000	0.41 ± 0.20
S	Malathion	1000	0.57 ± 0.09
R 1	NBD-Cl	270	0.61 ± 0.11
R 2	NBD-Cl	270	0.41 ± 0.15
S	NBD-Cl	270	0.58 ± 0.05

"SD"- Standard deviation

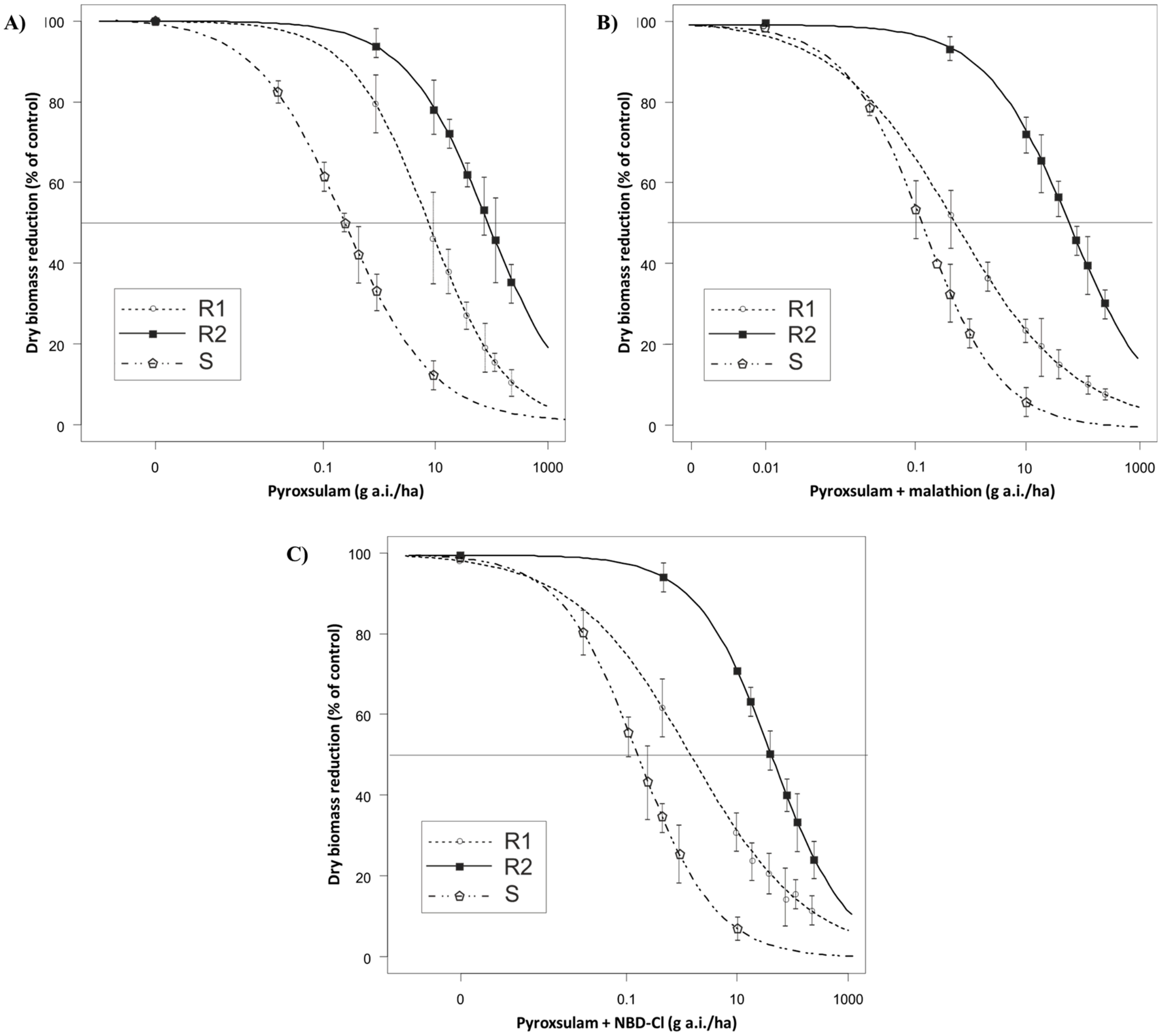

Figure S1. Fitted logarithmic dose-response curves for the *A. spica-venti* after application of A) pyroxsulam, B) pyroxsulam + malathion, and C) pyroxsulam + NBD-Cl.

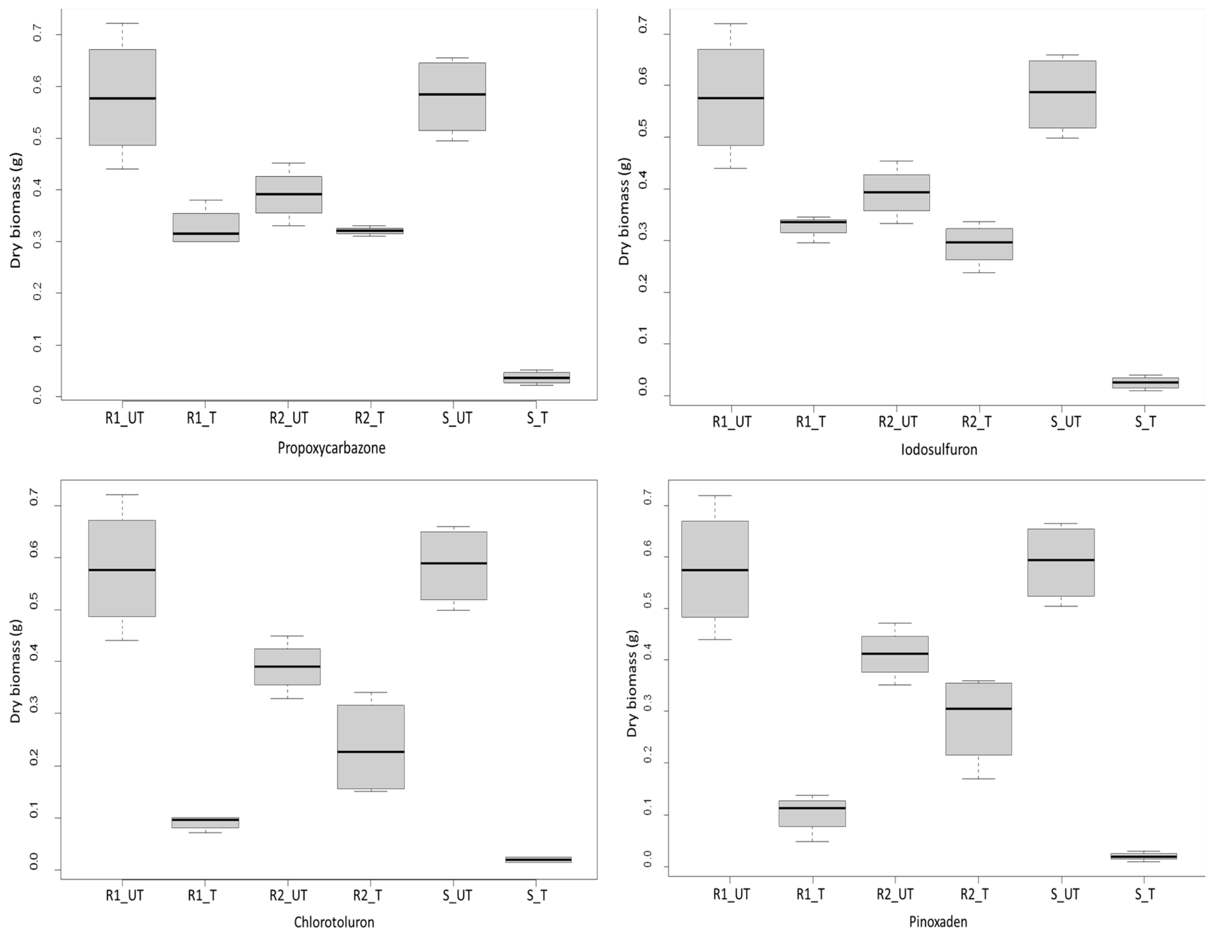

Figure S2. The dry biomasses of resistant (R) and the susceptible (S) biotype of A. spica-venti for cross and multiple resistance studies. “UT” refers to untreated and “T” refers to treated.
